# 4-Chloro-1,2-phenylenediamine induced structural perturbation and genotoxic aggregation in human serum albumin

**DOI:** 10.3389/fchem.2022.1016354

**Published:** 2022-09-19

**Authors:** Mohd Sharib Warsi, Safia Habib, Mohd Talha, Shifa Khan, Priyam Singh, Abdul Rouf Mir, Minhal Abidi, Asif Ali, Moinuddin

**Affiliations:** Department of Biochemistry, Faculty of Medicine, Jawaharlal Nehru Medical College, Aligarh Muslim University, Aligarh, India

**Keywords:** hair dyes toxicity, human serum albumin (HSA), 4-chloro-1,2-phenylenediamine, protein aggregation, genotoxicity

## Abstract

4-Chloro-1,2-phenylenediamine (4-Cl-OPD) is a halogenated aromatic diamine used as a precursor in permanent hair color production. Despite its well-documented mutagenic and carcinogenic effects in various *in vitro* and *in vivo* models, its role in fibrillar aggregate formation and their genotoxic effect in therapeutic proteins has received less attention. The significance of human serum albumin (HSA) arises from its involvement in bio-regulatory and transport processes. HSA misfolding and aggregation are responsible for some of the most frequent neurodegenerative disorders. We used various complementary approaches to track the formation of amyloid fibrils and their genotoxic effect. Molecular dynamics study demonstrated the complex stability. The impact of 4-Cl-OPD on the structural dynamics of HSA was confirmed by Raman spectroscopy, X-ray diffraction, HPLC and SDS-PAGE. Fibrilllar aggregates were investigated using Congo red assay, DLS, and SEM. The genotoxic nature of 4-Cl-OPD was confirmed using plasmid nicking assay and DAPI staining, which revealed DNA damage and cell apoptosis. 4-Cl-OPD provides a model system for studying fibrillar aggregation and their genotoxic potential in the current investigation. Future studies should investigate the inhibition of the aggregation/fibrillation process, which may yield valuable clinical insights.

## 1 Introduction

Since the birth of civilization, humans have been known to use cosmetics to change their hair colour, primarily among the female population (Harrison and Sinclair, 2003). Throughout human history, many people have wanted to change the appearance of their hair since it was a means to distinguish their social rank. The prominent market for permanent hair dyes, indicated by the growth of hair salons and their ability to apply temporary or permanent colour changes to hair while satisfying desires for beauty, fashion, and a younger appearance (Dias, 2015). One form of hair dye commonly used is oxidative or permanent hair colorant, which combines a dye precursor (such as an aromatic diamine) with an oxidizing agent (e.g., hydrogen peroxide). 4-Chloro-1,2-phenylenediamine (4-Cl-OPD), a halogenated aromatic diamine utilized in permanent hair colour ingredients, is one such dye is therefore worthy of investigation. Halogenated aromatic diamine, one of the ingredients in permanent hair dye formulations, has the potential to enter skin. These halogenated aromatic diamines can undergo successive oxidation and self-conjugation events that lead to the formation of an unstable primary quinonediimine intermediate, dimerization and trimerization products, and ultimately Bandrowski’s base (BB) (Jenkinson et al., 2009). Following the invention of the Ames test for mutagenicity, the first evidence that hair dye components can be hazardous to health, 169 marketable hair dye formulation were evaluated, with 150 of them mutagenicity positive (Ames et al., 1975). As a result, some hair dye chemicals were banned in the 1970s (Baan et al., 2008).

Permanent hair dyes are widely used in the United States, with over 80% of the market share (Lind et al., 2005). According to the US Environmental Protection Agency, around 15 million people are potentially exposed to hair dye components due to personal usage or through the application of hair dyes to others (Wang et al., 2008). 4-Cl-OPD caused hepatocellular carcinomas, papillomas, and urinary bladder carcinoma in mice and rat (Weisburger et al., 1980). According to epidemiological studies, hair dye addicts and hairdressers had a higher risk of bladder carcinoma, non-lymphoma, Hodgkin’s multiple myeloma, and hematological malignancies (Thun et al., 1994; Andrew et al., 2004; Rauscher et al., 2004). Carcinogenicity, mutagenicity, and cytotoxicity should all be considered when assessing the toxicity of chemicals, including colorants and their ingredients (Zhu et al., 1991; Zhu et al., 2005). For years, toxicologists and epidemiologists have been concerned about the potential carcinogenicity of hair dye chemicals (Burnett et al., 1980; Van Duuren, 1980). However, multiple investigations have revealed their potential toxicity to persons and the environment, necessitating the establishment of rules for their safety assessment, usage, disposal, and advising markers stating such hazards (Van Duuren, 1980; Wang et al., 2008).

Regarding the protein and hair dye relationship, Jenkinson *et al.* investigated the selective para-Phenylenediamine (PPD) modification of HSA at Cys34 and immunological response against PPD modified protein complex (Jenkinson et al., 2010). Though it is necessary to investigate structural and functional relationship between 4-Cl-OPD and HSA, it is thought that 4-Cl-OPD selective reactivity towards different amino acid residues such as lysine, cysteine, tyrosine, and tryptophan may play a role in the formation of structural alterations and fibrillar aggregate. Existing research on HSA modification by hair dye components fails to describe structural intermediates and fibrillar aggregates. HSA was chosen as the model for the study because it: (a) continues to maintain colloid osmotic pressure; (b) binds as well as transports a broad array of metabolites, including steroids, bilirubin, fatty acids, tryptophan, and hemin (Sadeghzadeh et al., 2020; Sharifi-Rad et al., 2020; Khashkhashi-Moghadam et al., 2022); (c) will provide an amino acid source during periods of malnutrition; and (d) acts as an antioxidant by scavenging free radicals (Burczynski et al., 1995; Pascolo et al., 1996; Bhattacharya et al., 2000; André et al., 2003; Zunszain et al., 2003). The exceptional ability of HSA to interact with the ligands is owing to the availability of multiple binding sites that can provide vital information on ligands pharmacological/toxicological activities, biotransformation, biodistribution, and other properties (Khan et al., 2020; Shahnawaz Khan et al., 2020; Khan et al., 2021a). Since, it has been shown that the binding of ligands to HSA can drastically alter the distribution and unbound concentration of different ligands (Bertucci and Domenici, 2002). The interaction of different chemicals with HSA results in the development of stable complexes, which can significantly impact the distribution and metabolism of blood-borne chemicals (Nišavić et al., 2018).

Aberrant interactions have been postulated to underpin the toxicity associated with protein aggregates in many neurodegenerative conditions such as Alzheimer’s, Parkinson’s, Creutzfeldt-Jakob disease, and others (Rajan et al., 2001; Chamani and Moosavi-Movahedi, 2006; Zare-Feizabadi et al., 2021; Maheri et al., 2022; Marjani et al., 2022). Proteins that have been post-translationally changed can be utilized as biomarkers to diagnose diseases or to evaluate therapeutic efficacy. A notable example of this is the quantification of glycated hemoglobin and glycoalbumin to diagnose and treat diabetes mellitus (Freitas et al., 2017; Dareini et al., 2020; He et al., 2021). Investigations into the impact of aromatic diamines on protein structures could lead to valuable clinical findings. As a result, it is critical to comprehend the process of aggregation/fibrillation prevention and develop effective inhibitors (Jenkinson et al., 2009).

In this study, HSA is used as a model for protein aggregation and its genotoxic implications in the presence of a hair dye component, 4-Cl-OPD, which is examined using a number of complementary approaches. We studied the Molecular dynamics (MD) simulation to understand the stability, binding mechanism and conformational dynamics of the 4-Cl-OPD-HSA complex. Raman spectroscopy, X-ray Diffraction (XRD), and High Performance Liquid Chromatography (HPLC) used to analyze structural changes in HSA. Congo red assay, Dynamic light scattering (DLS), electron microscopy, and SDS-PAGE were used to analyze albumin aggregates. Furthermore, we investigated the genotoxicity of aggregated HSA using a plasmid nicking assay and DAPI staining. Hence, this research reveals for the first time the role of 4-Cl-OPD, an ingredient of permanent hair color formulations, in the formation of fibrillar aggregates of HSA and its genotoxic effects.

## 2 Materials and methods

### 2.1 Chemicals

HSA (fatty acid free, 99%), 4-chloro-orthophenylenediamine (4-Cl-OPD), sodium dodecyl sulphate (SDS), sodium azide, low melting agarose, ethiduim bromide (EtBr), and dialysis tubing were purchased from Sigma Chemical Company, St. Louis, MO, United States. Acrylamide bisacrylamide, ammonium persulphate and N,N,N,N-tetramethylethylenediamine (TEMED) were purchased from Bio-Rad Laboratories, United States. All chemicals used in this study were of the highest analytical grade available. Deionized water was generated by a Direct-Q 3 UV water system from Merckmillipore, United States. Phosphate buffer saline (PBS) (10 mM) buffer solution was used to keep the pH of the solution at 7.4.

### 2.2 Modification of HSA

HSA was modified according to a published procedure (Warsi et al., 2021). Briefly Under sterile conditions, HSA (15 µM) was incubated with 4-Cl-OPD (200 µM) for 24 h in 10 mM PBS, pH 7.4 at 37°C in PBS. After incubation, native HSA and 4-Cl-OPD modified HSA were extensively dialyzed in PBS. All the measurements were carried out at room temperature.

### 2.3 Molecular dynamic (MD) simulations

The molecular docked conformation of the HSA-4-Cl-OPD complex with the lowest binding energy was chosen for molecular dynamics simulations (Hornak et al., 2006). GROMACS-2018.1 with the amber99 s b-ildn force field was used to perform the molecular dynamics (Ren et al., 2017). The topology of 4-Cl-OPD was developed using AmberTools19. The protein and complex were solvated in a triclinic box. The root mean square deviations (RMSD) and root mean square fluctuations (RMSF), radius of gyration (Rg), solvent accessible surface area (SASA), and energies were calculated using the GROMACS programme.

### 2.4 Raman spectroscopy

The Raman spectrum was performed on Raman InVia Reflex (Renishaw, United Kingdom) spectrometer using a laser of 785 nm wavelength. The laser power at the sample measuring was 5% and the WiRE spectral acquisition wizard generated a single scan measurement. The exposure time was set to 30.000s. Each spectrum was adjusted to account for the detecting system non-uniform spectral response; Renishaw Centrus 1C4A91 detector was applied to smooth the spectra.

### 2.5 X-ray diffraction (XRD) studies

The studies were carried out using a Rigaku Minifax X-ray-diffractometer with Ni-filter (Cu- Ka radiation l = 1.54186 A°) in the 2θ range of 5-70° with a scan rate of 8°/min. For XRD studies, native HSA and 4-Cl-OPD modified HSA samples were lyophilized. The peak positions, widths intensities, and shapes provided valuable information about the structure of the samples.

### 2.6 High performance liquid chromatography (HPLC) analysis

Acid hydrolysis of native HSA and 4-Cl-OPD modified HSA was carried out in 6 N HCl at 110°C for 24 h. The hydrolyzed samples were then subjected to ultrafiltration through 0.42 µM Milex filter, and analyzed on analytical HPLC (Shimadzu model LC-20AD) that runs on Isocratic mode. C-18 column and SPD-20 A UV-VISIBLE detector was used. 20 µl of each sample was loaded with the help of syringe, and HPLC grade 70% acetonitrile solvent was used as mobile phase with flow rate of 0.5 ml/min.

### 2.7 SDS-PAGE analysis

Native HSA and 4-Cl-OPD modified HSA samples were loaded on a 10% SDS polyacrylamide gel (Laemmli, 1970). 10 µg of protein sample was loaded into the gel wells, and electrophoresis was done at 60 V for 3 h with Coomassie Brilliant Blue staining (R-250). Imaging was done with the AlphaImager Gel imaging equipment.

### 2.8 Congo red (CR) binding assay

The binding of CR to the cross-structure of protein can be detected by measuring amyloid aggregates in solution (Cao et al., 2004). Before use, a fresh solution of CR in 10 mM PBS (pH 7.4) was filtered through a 0.2 μm filter. At room temperature, native HSA and 4-Cl-OPD modified HSA samples were incubated with CR at a molar ratio of 1:2 for 30 min. CR binding was measured spectroscopically in the 400–600 nm range.

### 2.9 Dynamic light scattering (DLS) measurements

The measurements were performed using DynaPro-TC-04 dynamic light scattering equipment (Protein Solutions, Wyatt Technology, Santa Barbara, CA) equipped with a temperature-controlled microsampler. Native HSA (15 µM) and 4-Cl-OPD (200 µM) modified HSA samples were stored at 37°C for 24 h. After spinning for 10 min at 10,000 rpm, the samples were filtered serially via 0.22 and 0.02 µm Whatman syringe filters directly into a 12 µl quartz cuvette. The mean hydrodynamic radius (R_*h*_) was analyzed at an optimum resolution using Dynamics 6.10.0.10 software. The R_*h*_ was calculated using the Stokes-Einstein relationship and autocorrelation analysis of scattered light intensity data based on translation diffusion coefficient:

$$R_{h}=\frac{ĸT}{6\pi \eta D\frac{25℃}{\omega }}$$

where R_*h*_ is the hydrodynamic radius, *k* is the Boltzmann constant, *T* is the temperature, *η* is water viscosity, and *D* is the diffusion coefficient.

### 2.10 Scanning electron microscopy (SEM)

SEM was used to examine the microstructures of native HSA and 4-Cl-OPD modified HSA. On cellulose ultra-filtration membranes, dried samples (5 µM) were adsorbed (Talha et al., 2021). They were gold-coated and mounted on stainless-steel grids with a carbon collodion coating over them. Electron micrographs were taken on JSM-6510LV (JEOL JAPAN) at 5000X resolution.

### 2.11 Plasmid nicking assay

For Plasmid nicking assay was performed following the method described by Tabrez and Ahmad (Tabrez and Ahmad, 2011). Here, covalently closed pBR322 (0.5 μg) in PBS served as control and pBR322 treated with the 4-Cl-OPD modified HSA was used as test sample, methyl methane sulphonate (MMS) was taken as positive control. Electrophoresis was performed on 1% agarose gel for 2 h at 50 mA.

And the samples were stained with ethidium bromide (0.5 mg/ml) and the gel was visualized by AlphaImager Gel documentation system (Protein Simple, United States) and photographed simultaneously.

### 2.12 DAPI staining

Human peripheral blood was drawn via venipuncture in vacuum tubes containing 0.1 mM EDTA in order to extract lymphocytes. According to the manufacturer’s instructions, peripheral blood cells were separated using a density gradient contained in the reagent Histopaque 1077 (Sigma-Aldrich) under sterile conditions. Utilizing Trypan blue (1%), lymphocytes were counted in a neubauer chamber following centrifugation. All experiments save those specifically indicated therein used an equal amount of lymphocytes (Alam et al., 2015).

Nuclear fragmentation and distortion in apoptosis were studied using DAPI, a blue-fluorescent DNA stain that binds to AT regions of dsDNA (Abdullah et al., 2018). In a 1:1 ratio, DAPI (20 mg ml^−1^) was added to protein samples and incubated for 5 min at room temperature. Fluorescence microscope (Carl Zeiss) was used to capture images after staining.

## 3 Results

### 3.1 Molecular dynamics (MD) simulation

MD simulations are widely used to estimate the kinetics of ligand binding mechanisms to serum albumin under certain solvent conditions. We performed MD simulations of free HSA and the HSA-4-Cl-OPD complex for 100 ns.

#### 3.1.1 Root mean square deviations (RMSD) and root mean square fluctuations (RMSF)

**FIGURE 1:**
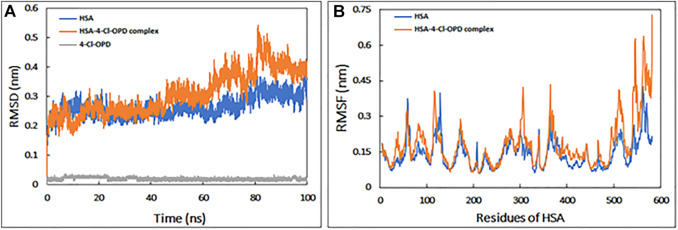
**(A)** Root mean square deviation (RMSD) HSA, HSA-4-Cl-OPD complex, and 4-Cl-OPD as a function of simulation time **(B)** Root mean square fluctuation (RMSF) of HSA and HSA-4-Cl-OPD complex.

The RMSD is commonly used to access the dynamic stability of systems; it represents a global measure of protein fluctuations. As shown in Figure 1A, the majority of the RMSD values fluctuate between 0.2 and 0.4 nm, whereas the RMSD of 4-Cl-OPD remained smooth throughout the simulation time. Lower RMSD values (<0.5 nm) for both HSA and the HSA-4-Cl-OPD complex indicate that the 4-Cl-OPD is stable in the HSA binding pocket. The RMSD of HSA during simulation indicates that the protein conformation changes only slightly after binding with 4-Cl-OPD.

RMSF of backbone of each amino acid residue of HSA and HSA-4-Cl-OPD complex was calculated from the trajectory as shown in Figure 1B. Even in the presence of 4-Cl-OPD, the RMSF of nearly all residues was less than 0.4 nm, showing that HSA was stable.

#### 3.1.2 Radius of gyration (Rg) and solvent accessible surface area (SASA)

**FIGURE 2:**
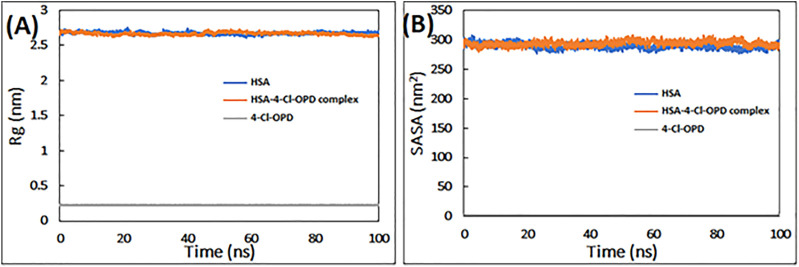
**(A)** Radius of gyration (R_g_) of HSA, HSA-4-Cl-OPD complex, and 4-Cl-OPD as a function of simulation time **(B)** Solvent accessible surface area (SASA) of HSA, HSA- 4-Cl-OPD complex, and 4-Cl-OPD as a function of simulation time.

To determine the requisite compactness and stability, Rg of HSA in the absence and presence of 4-Cl-OPD as a function of time was computed. As shown in Figure 2A The Rg of the HSA-4-Cl-OPD complex remained relatively constant throughout the simulation period, with an average value of 2.69 nm. Similarly, Rg of 4-Cl-OPD remained constant until 100 ns of simulation, indicating that 4-Cl-OPD did not undergo significant conformational changes.

Calculating variations in the SASA as a function of time was used to further investigate the complex stability. The data in Figure 2B clearly reveals that the SASA of HSA and HSA-4-Cl-OPD complexes changed very little during the course of the simulation, and that the SASA of 4-Cl-OPD stayed unchanged until the last 100 ns of the simulation. As a result, the stability of the HSA-4-Cl-OPD complex has been further confirmed.

### 3.1.3 Free energy land scape (FEL) and Ramachandran plot

**FIGURE 3:**
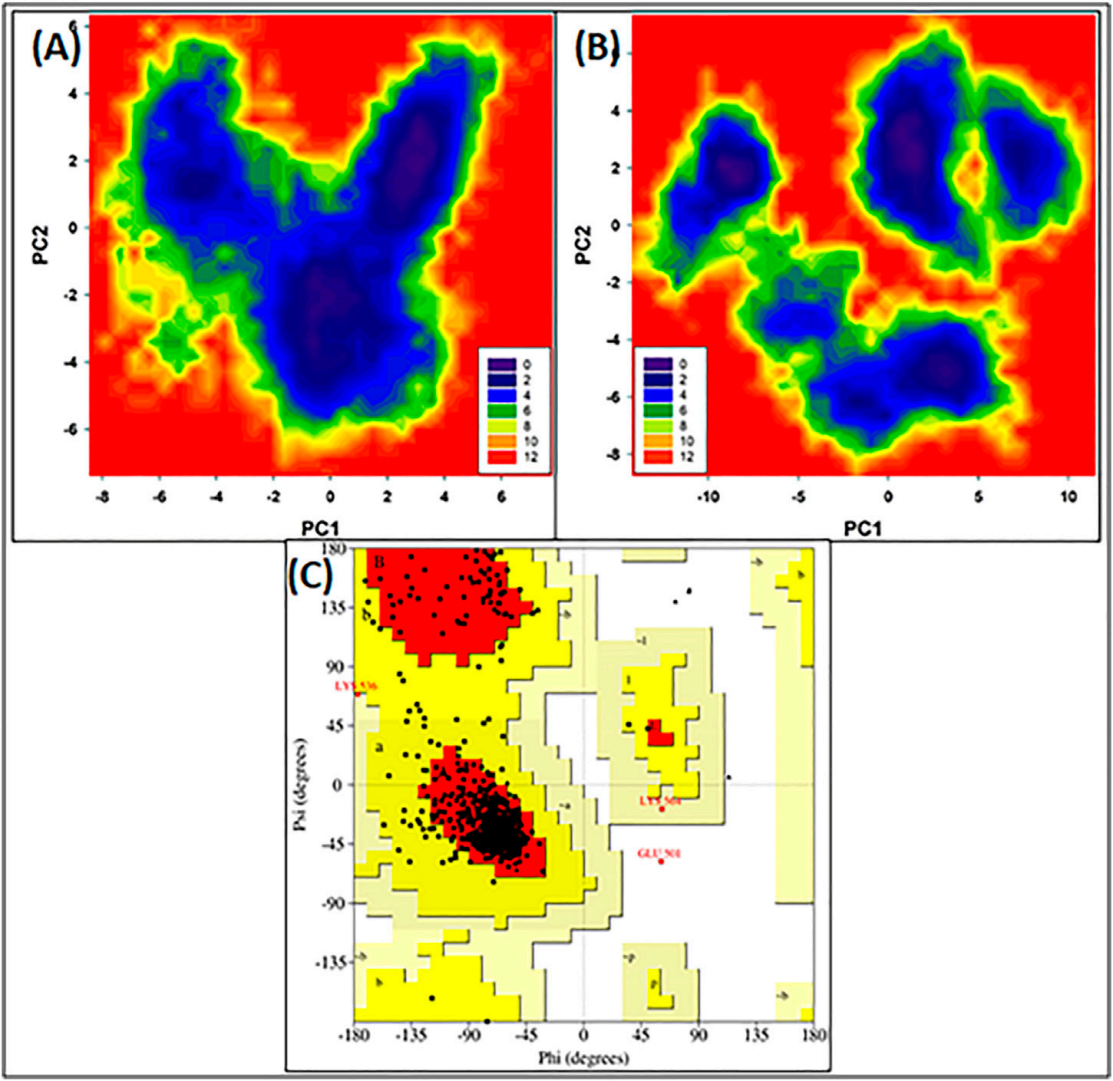
**(A)** Free energy land scape plot of HSA **(B)** Free energy landscape plot of HSA-4-Cl-OPD. **(C)** Ramachandran plot of energy minima structure of HSA-4-Cl-OPD complex.

Figures 3A,B illustrate the FEL values for free HSA and HSA-4-Cl-OPD complex. In the global free energy minimum region, there was just one primary free energy well/basin for the free HSA, indicating that only one stable conformational state resided within this well. At the same time, the HSA-4-Cl-OPD complex have three primary free energy wells indicating HSA-4-Cl-OPD complex spanned larger ranges of PC1 and PC2 and exhibited a more rugged free energy surface than free HSA and also showed larger number of local free-energy minima.

The phi (ϕ) and psi (ψ) distributions of the Ramachandran plot in Figure 3C showed that 100% of the residues were present in favored and allowed regions, confirming that the structure is comparable to a good homology model.

### 3.2 Structural characterization

#### 3.2.1 Raman spectroscopy

**FIGURE 4:**
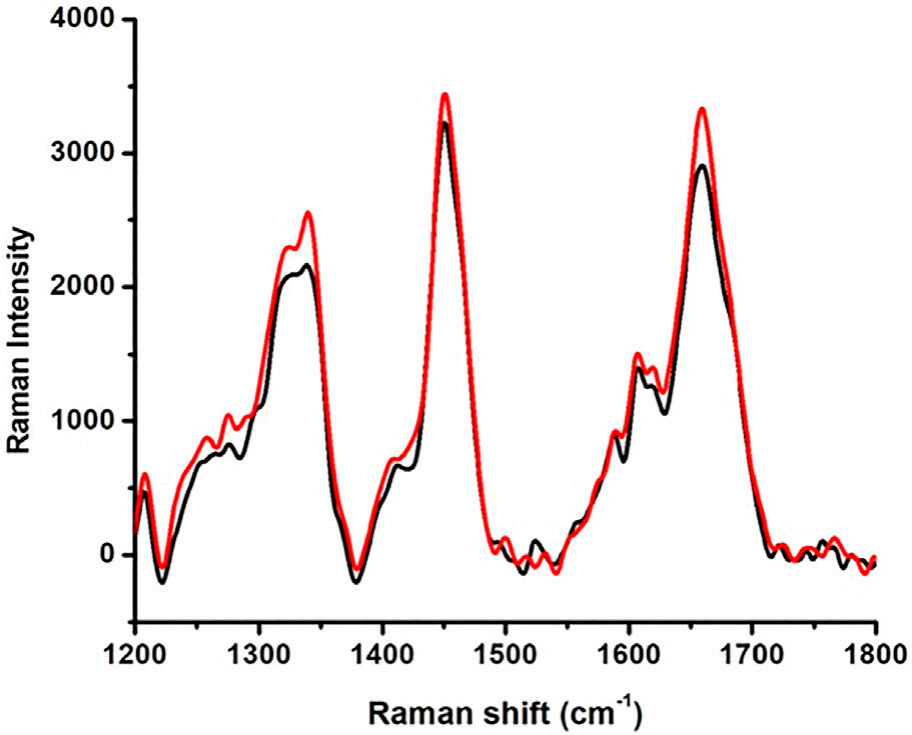
Raman spectroscopy native HSA (black line) and 4-Cl-OPD modified HSA (red line).

The Raman spectra of native HSA and 4-Cl-OPD modified HSA are shown in Figure 4. The 1655 cm^−1^ Amide-I band is a distinctive feature of *α*-helical (secondary conformation), the polypeptide backbone, arising mostly from peptide C=O stretching vibration. An increase in the intensity of this band would suggest a change in HSA secondary structure. Furthermore, increase in shoulder intensity between 1200 and 1400 cm^−1^ indicates a change in the tryptophan environment.

#### 3.2.2 X-ray diffraction (XRD) studies

**FIGURE 5:**
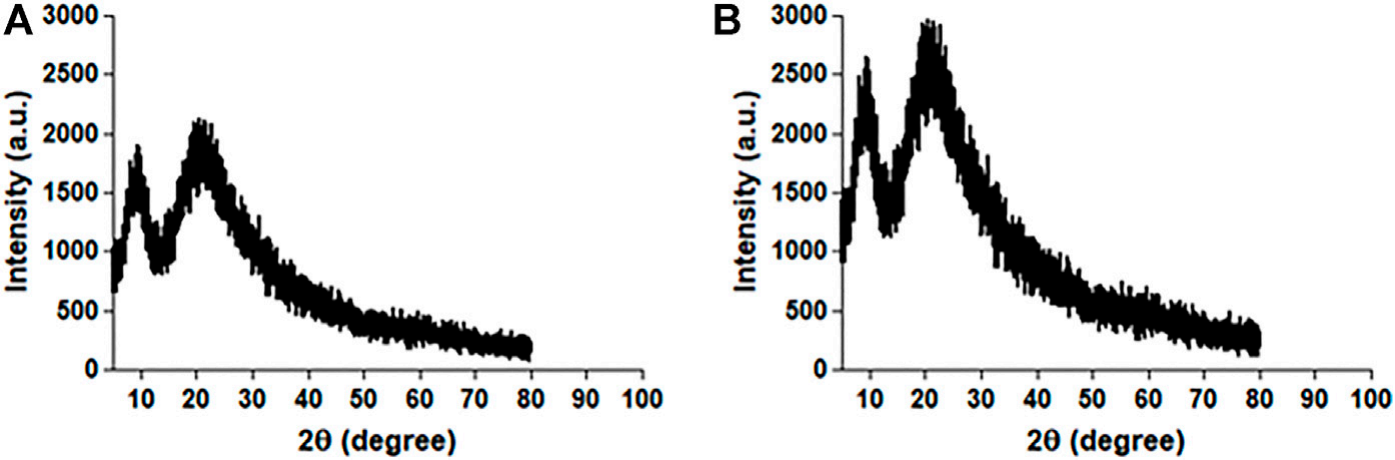
XRD pattern of **(A)** native HSA and **(B)** 4-Cl-OPD modified HSA.

Figure 5 shows an XRD plot of scattering intensity vs scattering angle (2θ) of native HSA and 4- Cl-OPD modified HSA. Both native HSA and 4-Cl-OPD modified HSA showed two distinct diffraction peaks in the crystalline region (2θ = 10.11°) and the amorphous region (2θ = 24.65°). The differences were seen in the scattering intensities, with the 4-Cl-OPD modified HSA having higher intensities than the native HSA. This is due to the formation of complex structures in case of 4-Cl-OPD modified HSA.

#### 3.2.3 High Performance Liquid Chromatography (HPLC)

**FIGURE 6:**
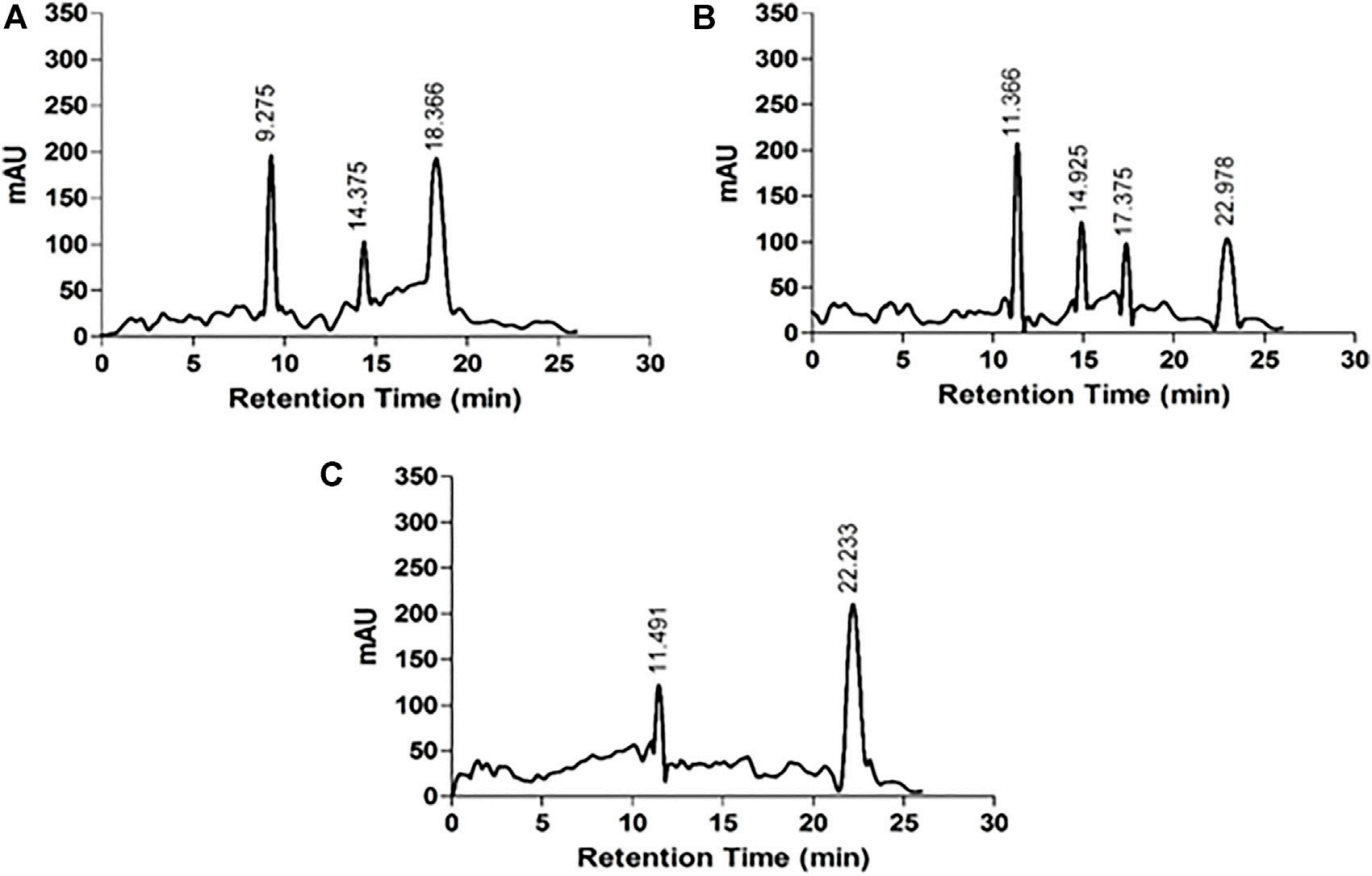
HPLC chromatograms of acid hydrolysates **(A)** native HSA **(B)** 4-Cl-OPD modified HSA and **(C)** l-cysteinesulfinic acid.

The HPLC chromatograms of acid hydrolyzed native HSA, 4-Cl-OPD modified HSA, and l-cysteine Sulfinic Acid used as a standard are shown in Figure 6. l-cysteinesulfinic acid is identified by a peak in the acid hydrolysate of 4-Cl-OPD modified HSA with a retention durations of 11.366 and 22.978 min. It showed that when native HSA is modified in the presence of 4-Cl-OPD, a structural intermediate called l-cysteinesulfinic acid is formed.

#### 3.2.4 SDS-PAGE analysis

**FIGURE 7:**
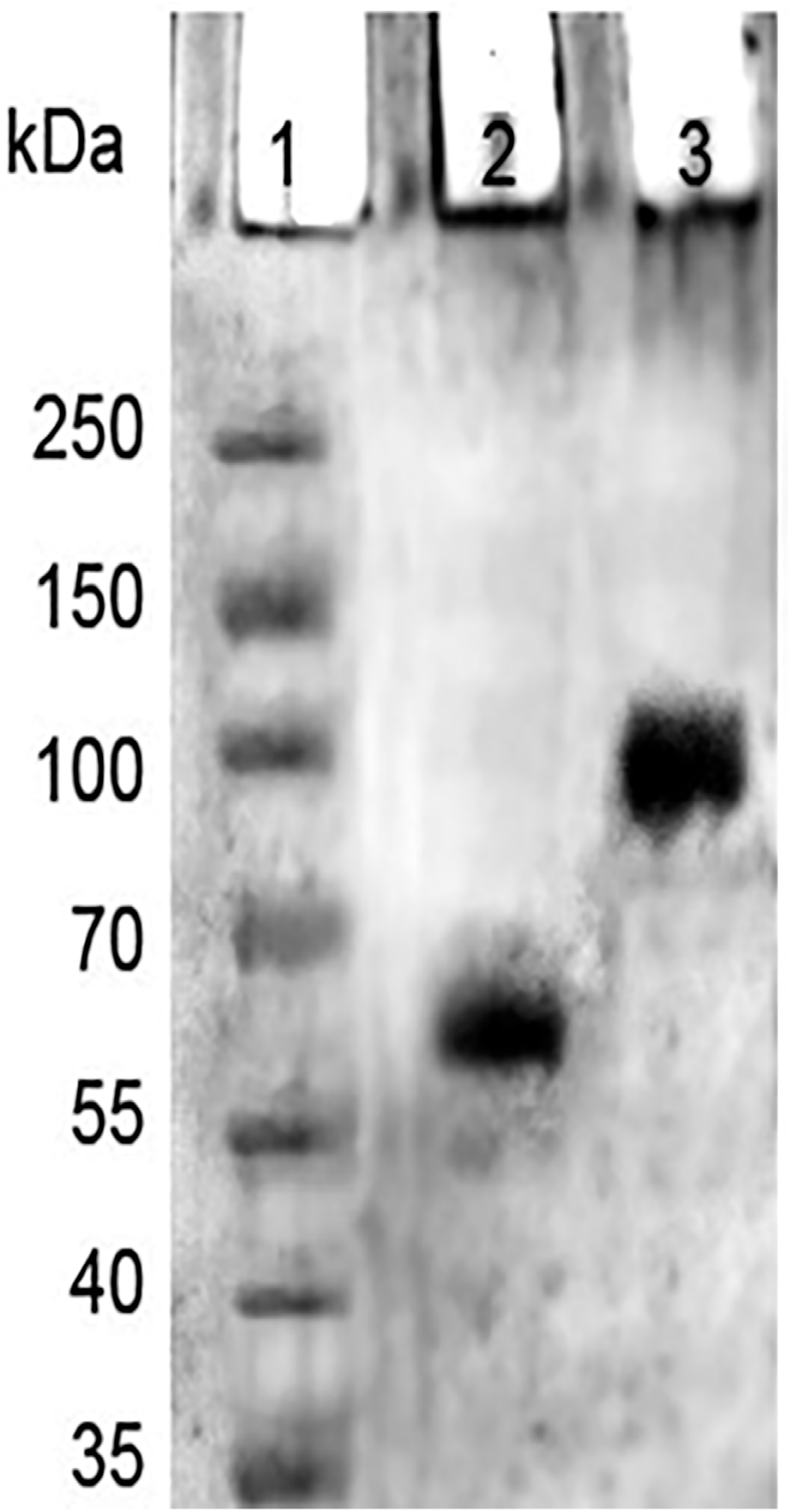
SDS-PAGE of markers (Lane1), native HSA (Lane 2), and, 4-Cl-OPD modified HSA (Lane 3).

In SDS PAGE analysis, in refrence to the marker protein, native HSA showed a noticeable single band, but 4-Cl-OPD modified HSA showed a decreased electrophoretic mobility and increased band width, as seen in Figure 7. This finding supports the formation of increased molecular weight cross-links in 4-Cl-OPD modified HSA.

### 3.3 Characterization of aggregates

#### 3.3.1Congo red (CR) assay

**FIGURE 8:**
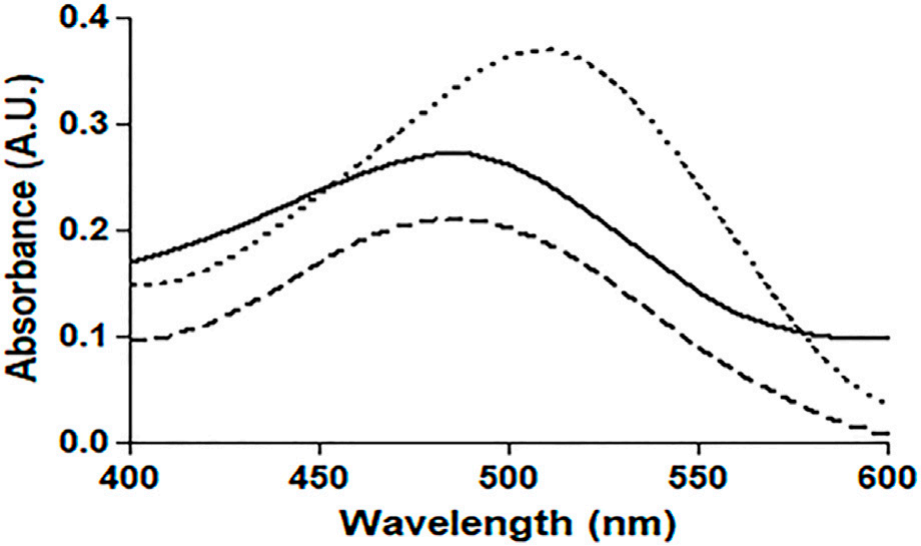
Absorbance profile of native HSA (**―**), 4-Cl-OPD modified HSA (۔ ۔ ۔),and Congo red alone(⁃ ⁃ ⁃).

The absorption profile of CR, which is a non-fluorescent dye, confirms its binding to protein fibrillar aggregates. Under our experimental conditions, binding of CR to native HSA and 4-Cl-OPD modified HSA produced a characteristic apple green birefringence and a red shift in the dye (λ_max_ from 490 to 520 nm), as seen in Figure 8. The CR spectrum was not significantly altered or red shifted after incubation with native HSA. In the 4-Cl-OPD modified HSA, the results support the formation of aggregates.

#### 3.3.2 Dynamic light scattering (DLS) measurements

**FIGURE 9:**
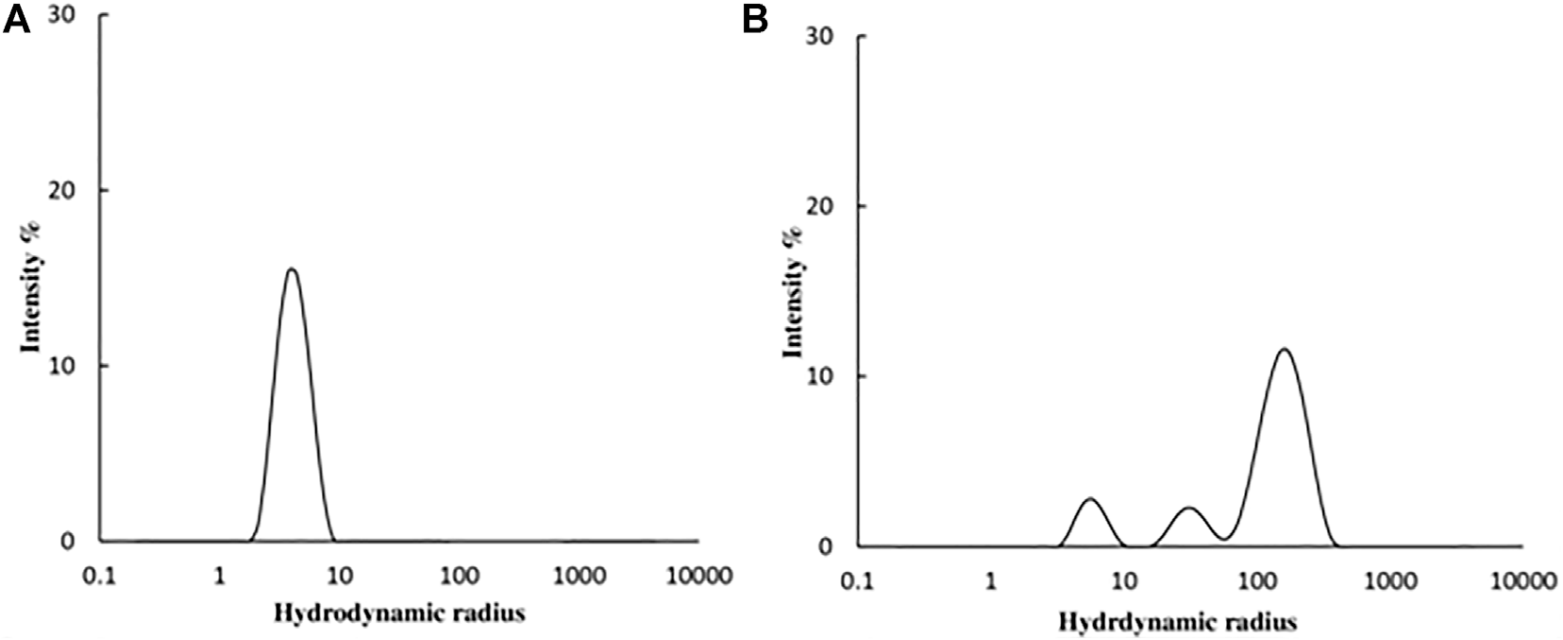
DLS profile of **(A)** Native HSA **(B)** 4-Cl-OPD modified HSA for determining hydrodynamic radii.

DLS studies were carried out to confirm the presence of aggregates. The hydrodynamic radius (R_*h*_) of native and 4-Cl-OPD modified HSA was investigated. As shown in Figures 9A, B, the R_*h*_ of native HSA was found to be 4.2 ± 1.2 nm, whereas R_*h*_ of 4-Cl-OPD modified HSA was found to be to 165.2 ± 59.8 nm, 32.2 ± 8.5 nm and 5.8 ± 1.3 nm. The increase in *R*_*h*_, signifies the presence of aggregated assemblies due to a reduction in intramolecular interactions in the presence of 4-Cl-OPD.

#### 3.3.3 Scanning electron microscopy (SEM) analysis

**FIGURE 10:**
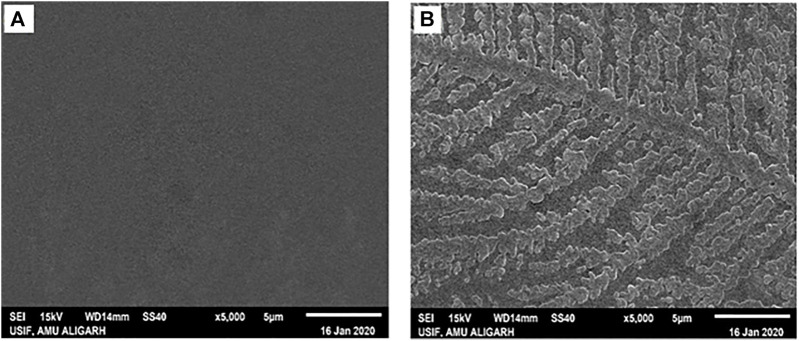
Scanning electron micrographs of **(A)** native HSA **(B)** 4-Cl-OPD modified HSA.

SEM was used to get an insight into the properties of species generated when HSA was incubated with 4-Cl-OPD for 24 h. Figure 10A depicts the SEM analysis of native HSA, while Figure 9B depicts the SEM analysis of 4-Cl-OPD modified HSA. As seen in Figure 9B, 4-Cl-OPD modified HSA induces the formation of visible fibrillar aggregates. Thus, it is clear that 4-Cl-OPD induces the formation of fibrillar aggregates.

### 3.4 Genotoxicity of HSA aggregates

#### 3.4.1 Plasmid nicking assay

The plasmid pBR322 was damaged in terms of loss of supercoiled DNA or the formation of single-strand breaks (SSBs) and double-strand br eaks (DSBs). In the n-CT-DNA, two major bands and one difused band correspond to the three forms. Form I- supercoiled molecules with no breaks, Form II- relaxed circular DNA, nicked circular and molecules with multiple SSBs, Form III- linear molecules of pBR322, linear molecules with one SSB, linear molecules with multiple SSBs.

**FIGURE 11:**
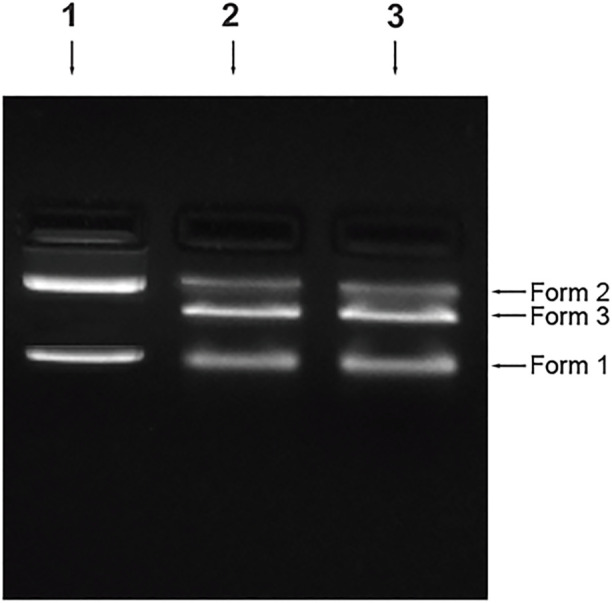
Plasmid nicking assay Control (Lane 1), 4-Cl-OPD modified HSA (Lane 2), positive control-MMS (Lane 3).

As shown in Figure 11, treatment of the plasmid with the 4-Cl-OPD modified HSA results in loss of Form I and an increase in Form III can be seen which signifies generation of SSBs first (Form II) which are then converted to DSBs, later generating linear plasmid DNA molecules.

#### 3.4.2 DAPI staining

**FIGURE 12:**
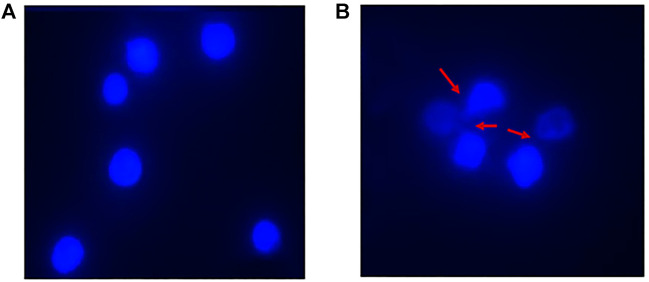
DAPI stained fluorescent images of **(A)** Native HSA **(B)** 4-Cl-OPD modified HSA (wherein red arrows show nuclear fragmentation and distortion).

The DAPI (4′,6-diamidino-2-phenylindole) staining method, indicates nucleus deformation and fragmentation. It revealed round nuclei in cells treated with native HSA as shown in Figure 12A. However, fragmentation and deformation of nuclei were seen in the cells treated with 4-Cl-OPD modified HSA as shown in Figure 12B.

## 4 Discussion

For many years, toxicologists and epidemiologists have been concerned about the potential carcinogenicity of hair dye chemicals (Marzulli et al., 1978; Nohynek et al., 2004). Previous research has suggested that PPD targets at Cys 34 of HSA and have mainly shown covalent alteration of HSA at cysteine residue and the formation of protein-hapten complexes that result in the generation of immunogenic complexes (Jenkinson et al., 2010). The impact of 4-Cl-OPD on cellular redox status, DNA integrity, and cytotoxicity have been investigated (Khan et al., 2021b; Warsi et al., 2021). Thus, it is pivotal to investigate the impact of 4-Cl-OPD on HSA and identify the significant structural modifications in the protein that lead to the development of aggregates and their genotoxic effects. This research could lead to developing a new index for assessing the potential role of 4-Cl-OPD on HSA. In this study, we show that 4-Cl-OPD, a PPD derivative, has a great potential as a modifying agent against HSA, resulting in alterations in structural dynamics, characterization of fibrillar aggregates, and assessment of aggregate genotoxicity.

In this study, we explored binding interactions and their stability and the structural modifications of HSA in the presence of 4-Cl-OPD. Previous *in silico* studies reported that 4-Cl-OPD coupled at subdomain IB of HSA with binding energy as -5.1 kcal mol^−1^4-Cl-OPD interacted with Asp108 and Arg145 via hydrogen bonds and Ala194 and Arg197 of HSA showed interaction with 4-Cl-OPD via hydrophobic interactions. Moreover, Tyr148 and Ser193 formed van der Waals interaction with HSA. Binding affinity is influenced by non-covalent intermolecular interactions such as hydrogen bonding, electrostatic interactions, hydrophobic and Van der Waals forces between the two molecules. Understanding the binding affinity to ligands is significant while characterizing protein modifications. Earlier studies have reported characterization of the structural changes of HSA upon interaction with single-walled and multi-walled carbon nanotubes was done through spectroscopic and molecular modeling approaches (Kabiri et al., 2012; Hosseinzadeh et al., 2019). The binding constant values were found to determine the different binding affinity of two types of nanotubes to HSA that cause induction of different conformations in protein.

The reason for this gain in binding affinity is the fact that the increased protein-ligand interaction surface results in stronger van der Waals interaction. Binding energy, van der Waals interaction, hydrogen bonds, and hydrophobic interactions all have a role in the stability of HSA in the presence of 4-Cl-OPD. Existing studies also reports subdomain IB as one of the major drug binding regions of HSA having strong ligand recognition ability (Warsi et al., 2021).

Many studies have reported that the differences in binding energies and thermodynamic parameters obtained using experimental and computational methods. When we perform *in silico* environment, it gives us a theortical value of binding energy When we perform *in vitro*, many elements, such as H-bonding, polar-polar interactions, dipole forces, van der Waals forces, and even light, might affect the binding energy value. Water has an important role in the biological environment, especially in the protein matrix. Water has a crucial role in ligand binding thermodynamics, even in the environment of a lipophilic binding cavity (Pantsar and Poso, 2018). Ligand optimization *in vitro* may be achieved by displacing specific water molecules from the binding site (Michel et al., 2009). Moreover, water related H-bonding networks have a significant influence in the structure-activity relationship (Biela et al., 2013), and optimizing the ligand taking into account the surrounding water network may result in enhanced binding affinity and prolonged residence time (Krimmer et al., 2016). Molecular docking is currently unable to consider the impact of water and the dynamic nature of the binding (Ruiz-Carmona et al., 2017). Furthermore, easy application of water placement in docking is restricted because the water in the binding site is heterogenous and in different locations, an individual water molecule has restricted rotational freedom and H-bonding capabilities (Pantsar and Poso, 2018).

For detailed view, MD simulations were performed to examine the binding stability and mechanism of binding of HSA complex with 4-Cl-OPD. The RMSD of HSA during simulation suggests that the protein structure changes only slightly following interaction with 4-Cl-OPD. Lower RMSD values for both HSA and the HSA-4-Cl-OPD complex indicate that the 4-Cl-OPD is stable in the HSA binding pocket. During MD modelling, the RMSF of the HSA and HSA-4-Cl-OPD complex offers information about protein local structural mobility. Low residual variations in HSA residues indicate that only a slight change in HSA conformation occurs when it interacts with the 4-Cl-OPD (Soltanabadi et al., 2018). This fluctuation analysis demonstrated that even after complexation with 4-Cl-OPD, a similar pattern of fluctuation was detected in HSA residues. The Rg of both HSA and HSA-4-Cl-OPD complex remained nearly constant throughout the simulation. Under physiological conditions, it showed negligible conformational modifications in both forms (Qais et al., 2021). The stability was further studied by analyzing the changes in the SASA. The SASA of both structures displayed low variations, supporting the stability of the HSA-4-Cl-OPD complex. In the MD simulation, the Rg and SASA of the HSA-4-Cl-OPD complex remain constant throughout with no fluctuations, describing the compactness and stability of 4-Cl-OPD in the binding pocket of HSA. Free energy landscapes were also plotted to further explain the changes in protein-folding processes, as the HSA-4-Cl-OPD complex possessed a bigger, more “rugged,” and complicated free-energy surface than free HSA. The Ramachandran plot confirmed that HSA-4-Cl-OPD complex is comparable with good homology model (Zhu et al., 2011; Sang et al., 2017).

In Raman spectral studies, HSA reflected 31 Phe, 18 Tyr, and 1 Trp residues, which give rise to several Raman bands due to aromatic ring vibrations. The 1655 cm^−1^ Amide-I band is a distinctive feature of the polypeptide backbone secondary conformation, arising primarily from peptide C=O stretching vibration. An increase in the intensity of this band would indicate a change in the secondary structure of HSA (Torreggiani and Tinti, 2010). Furthermore, a shift in the Trp environment could be indicated by increasing shoulder intensity in the 1200–1400 cm^−1^ range. Because of the modification of native HSA by 4-Cl-OPD, we detected various biochemical changes in the mentioned spectrum regions (Chinnathambi, 2016). XRD investigations validated the amorphous and crystalline regions of native HSA and 4-Cl-OPD modified HSA. Increased peak intensities in the amorphous and crystalline regions of 4-Cl-OPD modified HSA demonstrated the formation of complex structures formation compared to native HSA. As a result, XRD findings confirmed significant structural changes in HSA after 4-Cl-OPD modification (Gong et al., 2011; Malik and Javed, 2021).

HSA antioxidant activity is another key factor; its function is thought to be due to its single exposed thiol group at Cys34, which can serve as a significant antioxidant in plasma (Nagumo et al., 2014). The acid hydrolysate of 4-Cl-OPD modified HSA showed a peak with a retention period of 22.50 min, which is typical of the l-cysteinesulfinic acid, used as a standard in HPLC analysis (Ida and Kuriyama, 1983). It showed that 4-Cl-OPD was selectively modified at Cys34 residue of oxidised HSA. Thus, an elevated level of oxidised HSA may compromise HSA function, mainly its free radical scavenging capability. Due to the oxidative modification of HSA by 4-Cl-OPD, SDS-PAGE analysis showed the formation of high molecular weight cross-links or aggregates in 4-Cl-OPD modified HSA compared to the native HSA (Talha et al., 2021). The presence of antigenic determinants in dye modified HSA adducts has been demonstrated, and these adducts have been linked to an immunological response in contact dermatitis (Jenkinson et al., 2009). Additionally, modified HSA becomes immunogenic in a number of disorders (Jyoti et al., 2016). Studies on immunogenicity of 4-Cl-OPD modified HSA constitute a different study which is underway within our lab.

Amyloid or amorphous protein aggregates are the outcome of oxidative stress-induced protein aggregation. Compared to amorphous aggregates, the amyloid state is highly organized (Weids et al., 2016). In the CR study, CR is extensively used in the field of amyloid fibril analysis, When CR interacts with amyloid fibrils, a shift in the UV absorbance from about 490 to 540 nm occurs. Furthermore, an apple green birefringence in polarized light and an induction of a circular dichroism between 300 and 600 nm can be observed for Congo Red in presence of amyloid fibrils. Congo Red is prone to self-assembly in water and has been suggested to bind to amyloids mainly as a supramolecular ligand (Naiki et al., 1989; Stopa et al., 1998; Stopa et al., 2003). Binding of CR to 4-Cl-OPD modified HSA resulted in a typical apple green birefringence and a characteristic red shift in UV absorbance from 490 to 520 nm, but native HSA showed no significant alteration or red shift (Frid et al., 2007). The red shift is caused by the expansion of the conjugated π-electron system of CR when it binds to amyloid aggregates (De Vasconcelos and Ximenes, 2015). A red shift of the absorption band toward 540 nm and an increase in absorption were together taken to be indicative of the formation of amyloid structures *in vitro* (Meehan et al., 2004; Yakupova et al., 2019). The CR molecules were found to aggregate into larger assembly which may be its forms in binding to amyloid fibrils and a cause in its red shift in UV/vis absorption (Cooper and Stone, 1998). Inouye and Kirschner, in their study showed that regular arrangement of histidines on the protofilament of amyloid fibril may act as a template for the end-to-end J-aggregate of CR molecules, which produces a red shift in UV/vis absorption. Had it been as H-aggregate, it would have reflected blue shift. In addition, they argued that CR likely binds electrostatically to the imidazolium side chains of histidine residues that are exposed at the surface of amyloid fibrils (Inouye and Kirschner, 2005; Wu et al., 2012). The findings suggest aggregate formation in 4-Cl-OPD modified HSA. To confirm our findings, we analyzed the kinetics of particle size distribution in native HSA as well as in 4-Cl-OPD modified HSA using DLS. The kinetics aggregation showed a gradual increase in particle size in 4-Cl-OPD modified HSA with three clearly distributed populations, R_*h*_ was found to be to 165.2 ± 59.8 nm, 32.2 ± 8.5 nm and 5.8 ± 1.3 nm. Thus, the DLS data confirmed our CR data, increased R_*h*_ indicate the presence of aggregated assemblies due to decreased intramolecular interactions (Chaturvedi et al., 2015). Increased R_*h*_ causes hydrophobic residues to be exposed, allowing them to interact with one another to establish intermolecular interactions and hence, aggregates of albumins (Arya et al., 2014; Ajmal et al., 2017). SEM analysis of the micro-architectural features and morphological characteristics of modified proteins revealed the formation of many forms of aggregation. Both SEM and DLS are available methods for analyzing various sizes of aggregates. However, their principles of measurement are different as the object size from DLS is calculated by diffusion coefficient, but SEM can directly observe the size of objects (Den Engelsman et al., 2011). Furthermore, DLS is capable of *in situ* monitoring under wet conditions, without staining, and at atmospheric pressure, while conventional SEM should be carried out with *ex situ* monitoring under high-vacuum conditions, using stained and dried samples. The images from SEM may include artifacts due to drying, metal staining, or coating (Senga et al., 2019). 4-Cl-OPD-mediated alterations resulted in the formation of large fibrillar aggregates in contrast to tiny granular shape-like structure in native HSA (Iram et al., 2013; Mir et al., 2015). The partial unfolding of the tertiary structure and conformational changes in the secondary structure are the causes of the protein aggregation/fibrillation phenomena (Juárez et al., 2009). Understanding the mechanism of aggregation/fibrillation prevention is crucial for designing effective inhibitors. Finally, we documented the genotoxic nature of aggregated HSA, the genotoxicity of 4-Cl-OPD modified HSA is due to the oxidative damage to DNA are by the production of reactive oxygen species. Plasmid nicking assay indicated the enhanced strands scission resulting in the formation of single-strand breaks in the plasmid (Tabrez and Ahmad, 2011). DAPI-treated cells showed increased nuclear fragmentation and distortion, indicating apoptosis in 4-Cl-OPD modified HSA compared to no fragmentation in native HSA (Atale et al., 2013). There is a growing body of evidence suggesting that oxidative stress plays a central role in genotoxicity (Gebel, 1997; Kitchin and Ahmad, 2003). This study examined the impact of 4-Cl-OPD on structural dynamics of HSA and characterization of genotoxic fibrillar aggregates.

## 5 Conclusion

The present study reveals that 4-Cl-OPD and HSA forms a strong and stable complex. Complex formation causes structural alteration and formation of aggregates in HSA that shows genotoxic effects *in vitro*. This study reporting that 4-Cl-OPD can cause aggregates formation.

This research has paved the way for a more in-depth understanding of the newly discovered effects of 4-Cl-OPD in the formation of protein aggregates, genotoxicity of aggregates and molecular basis of aggregate caused-disorders.

## Data Availability

The raw data supporting the conclusions of this article will be made available by the authors, without undue reservation.
